# Complete Genome Sequence of a Radioresistant Bacterial Strain, Deinococcus grandis ATCC 43672

**DOI:** 10.1128/MRA.01226-19

**Published:** 2019-11-07

**Authors:** Atsushi Shibai, Katsuya Satoh, Masako Kawada, Hazuki Kotani, Issay Narumi, Chikara Furusawa

**Affiliations:** a Center for Biosystems Dynamics Research (BDR), RIKEN, Suita, Osaka, Japan; b Project Ion Beam Mutagenesis, Department of Radiation-Applied Biology Research, Takasaki Advanced Radiation Research Institute, Quantum Beam Science Research Directorate, National Institutes for Quantum and Radiological Science and Technology, Takasaki, Gunma, Japan; c Faculty of Life Sciences, Toyo University, Itakura, Gunma, Japan; d Universal Biology Institute, Graduate School of Science, The University of Tokyo, Bunkyo-ku, Tokyo, Japan; Portland State University

## Abstract

Deinococcus grandis is a radioresistant bacterial species isolated from freshwater fish. In this article, we report the complete genome sequence of *D. grandis* strain ATCC 43672. This sequence is useful for comparative genomics to understand the traits of Deinococcus species and can be used as a reference in experimental genetics.

## ANNOUNCEMENT

*Deinococcus* is the most well-known radioresistant bacterial genus, and more than 70 species have been isolated so far (http://www.bacterio.net/deinococcus.html). The mechanisms and relevant proteins of their DNA repair systems have been intensively studied (1–5). Deinococcus grandis is a radioresistant bacterium isolated from freshwater fish in Japan, and a draft genome sequence of this species was previously reported (6), which only used short-read (∼400 bp) next-generation sequencing (NGS) technology. In this study, we report the complete genome sequence of *D. grandis*, obtained using a hybrid assembly strategy that combines short- and long-read technologies to generate *de novo* circular sequences.

*D. grandis* ATCC 43672 cells were grown in 10 ml of TGY medium (0.5% tryptone, 0.1% glucose, 0.5% yeast extract, and 0.1% KH_2_PO_4_) at 34°C for 2 days and then pelleted. We extracted the genomic DNA from the cells using the Wizard genomic DNA purification kit (Promega). Short-read library preparation for Illumina paired-end sequencing (2 × 300 bp) was performed with Nextera XT kit (Illumina) and sequenced on an Illumina MiSeq platform using the MiSeq reagent kit v3 with 600 cycles. A long-read library for Nanopore sequencing was prepared using a 1D^2^ sequencing kit (SQK-LSK308; Oxford Nanopore) without a fragmentation step. Then, a MinION device with a 1D^2^ flow cell (FLO-MIN107; Oxford Nanopore) was used to sequence the sample, and the Guppy v2.3.5 (Oxford Nanopore) software was used to call the bases. MiSeq sequencing generated 3,316,736 paired short reads. MinION sequencing generated 21,246 reads (0.14 Gb), with an average length of 6,448.5 bp.

A hybrid *de novo* assembly of short and long reads was performed using Unicycler v0.4.8 (7), with the default parameters, including a polishing step with Pilon v1.2.3 (8). Initially, we obtained five circular contigs and 30 linear contigs. Then, we conducted Nucleotide BLAST (v2.9.0+; https://blast.ncbi.nlm.nih.gov/Blast.cgi) analyses comparing these contigs. We found that one circular contig (1,029 bp) and 29 linear contigs were highly similar (>99% identity) to the subsequences of four unique circular contigs, suggesting that they are misassembled artifacts. Though there was the remaining one short linear contig (314 bp), we manually determined that the four unique circular contigs are the complete genome. The resulting circular contigs were additionally polished using Pilon.

The genome sequence of *D. grandis* ATCC 43672 was 4,013,039 bp, consisting of four circular contigs (3,241,502 bp, 389,567 bp, 373,915 bp, and 8,055 bp), and was GC rich (69.9%). The genome was automatically annotated using the DFAST pipeline (9), resulting in 3,977 coding sequences (CDSs). The third circular contig corresponds to three linear DNAs that were not linked in the previously reported draft genome (6). One of the circular contigs reported in the draft genome (accession number BCMS01000006) was lost this time. Since the largest CDS in the unfound contig is a phage tail protein, this circular DNA might be a mobile genetic factor, and as a result, it is thought that it was lost before this sequencing.

### Data availability.

The complete genome sequence of *D. grandis* ATCC 43672 was deposited as a set (a chromosome and three plasmids) in DDBJ/GenBank under accession numbers AP021849 to AP021852. The fastq files of the raw reads were deposited in the DDBJ Sequence Read Archive (DRA)/NCBI SRA under accession number DRA008993.
